# Radioactive iodine therapy strategies for distinct types of differentiated thyroid cancer: a propensity score–matched analysis

**DOI:** 10.3389/fendo.2023.1158581

**Published:** 2023-08-17

**Authors:** Honghao Guo, Ning Zhang, Yixuan Hu, Furong Zhang, Tao Huang, Na Shen

**Affiliations:** Department of Breast and Thyroid Surgery, Union Hospital, Tongji Medical College, Huazhong University of Science and Technology, Wuhan, China

**Keywords:** radioactive iodine (131 I) treatment, thyroid cancer (TC), differentiated thyroid cancer (DTC), survival, propensity score (PS) matching (PSM)

## Abstract

**Background:**

The management guidelines of radioactive Iodine (RAI) therapy for distinct types of differentiated thyroid carcinoma (DTC) were the same in clinical practice. However, in distinct types DTC, differences in RAI avidity and response existed and the effect of RAI therapy could not be equated.

**Methods:**

DTC patients’ data in SEER database were extracted to perform retrospective analysis. The differences between case group and control group were compared by chi-square tests. We used Kaplan-Meier statistics and Cox regression analyses to investigate cancer-specific survival (CSS). Propensity score–matched was performed to make 1:1 case-control matching.

**Results:**

105195 patients who receiving total thyroidectomy were identified in SEER database. Compared to papillary thyroid carcinoma (PTC) (52.3%), follicular thyroid carcinoma (FTC) (63.8%) and oncocytic carcinoma of thyroid (OCA) (64.4%) had higher rates of RAI therapy. In the multivariable Cox regression model, RAI therapy was independent prognosis factor in PTC but not in OCA and FTC. In subgroup analysis, RAI therapy could improve prognosis in PTC when gross extrathyroidal extension or lymph node metastases or early survival when distant metastases (DM) were presented. However, OCA and FTC patients with DM rather than regional lesions only could benefit from RAI therapy. High-risk patients receiving RAI therapy showed a better prognosis in PTC but not in OCA and FTC.

**Conclusion:**

RAI therapy was an effective treatment for DTC and should be considered individually in PTC, OCA and FTC patients. Our results provided further guideline for treatment selection in DTC.

## 1 Introduction

Thyroid cancer (TC) was the most common endocrine malignancy (1), affecting 567,000 cases worldwide, according to global cancer statistics (2). Differentiated thyroid carcinoma (DTC) originated from follicular epithelial cells and encompassed papillary thyroid carcinoma (PTC), follicular thyroid carcinoma (FTC) and oncocytic carcinoma of thyroid (OCA). PTC was the predominant type of TC accounting for 80-90% of cases, followed by FTC at 5-10%, and OCA at 3-4% (3–6). The 5th edition of the World Health Organization classification of tumors defined OCA as an invasive malignant follicular cell tumor consisting of at least 75% oncocytic cells, lacking the characteristic nuclear features of PTC or high-grade features (7). PTC had a higher incidence of regional lymph node metastases (LNM) due to its lymphatic permeation, while OCA and FTC were encapsulated tumors with capsular and/or vascular invasion that tended to spread hematogenously (7–9). Compared with PTC, OCA and FTC exhibited more aggressive clinicopathological features and worse prognosis (10–13).

Radioactive iodine (RAI) had been widely used in DTC patients after total thyroidectomy (TT) due to its targeting ability for thyroid cancer cells. American Thyroid Association (ATA) management guidelines recommended postoperative risk of recurrence risk stratification of DTC to guide RAI decision-making (1). ATA risk staging (TNM) was a relatively simple tool that contains vital clinical information on tumor size, lymph nodes and distant metastases, which could largely reflect the patient’s risk of post-operative recurrence and guide RAI therapy. However, differences in the biological behavior of PTC, FTC, and OCA had led to differences in response to RAI therapy. Most guidelines, including those of the ATA and National Comprehensive Cancer Network (NCCN) (14), incorporated PTC, OCA, and FTC into DTC for management and used the same strategy for RAI therapy. Inappropriate RAI treatment could lead to unnecessary complications, affecting quality of life and shortening survival (15). RAI avidity had been found to be independently correlated with pathological types of DTC with distant metastases (DM). In addition, there was no difference in RAI response between OCA and FTC, and both showed more disease progression after RAI compared to PTC (16). The role of RAI in FTC and OCA was still controversial with some studies suggesting it may improve survival outcomes while others had found no significant benefit (17). Previous researches revealed that RAI was not independently prognosis factor and could not improve cancer-specific survival (CSS) in OCA (18, 19). A retrospective study showed that minimally invasive FTC patients receiving RAI did not have better CSS than those without (20). Some retrospective studies considered that RAI could improve survival outcomes in OCA and FTC (21–24).

The objective of our study was to analyze the clinicopathological characteristics of three different types of DTC and compare the impact of RAI on CSS among them in order to refine the guidance for RAI in clinical practice. We conducted stratification analysis of DTC using ATA risk staging (TNM) and performed propensity score-matching (PSM) to control for potential confounders (1).

## 2 Materials and methods

### 2.1 Database and population

We identified patients diagnosed with PTC, OCA and FTC between 2004 and 2017 from the National Cancer Institute’s Surveillance, Epidemiology, and End Results (SEER) program. Publicly accessible data in SEER program were collected from 18 population-based central cancer registries. The third edition of International Classification of Diseases for Oncology (ICD-0–3) codes: 8050, 8260, 8290, 8330-8332, 8335, and 8339, 8340–8344, 8450 were included in our sample. The inclusion criteria were as follows: age older than 18 years and younger than 90 years, receipt of total thyroidectomy (TT), radiation exception of external radiation treatment, T stage exception of unknown and Tx, survival months greater than 0, and SEER cause-specific death classification exception of Dead (unknown or missing COD).

### 2.2 Study variables

Variables of interest included age at diagnosed (aged <55 and ≥55 years), sex, race (White, Black, and Other [American Indian/Alaska Native, and Asian or Pacific Islander]), T stage, N stage, M stage, ATA risk staging (low-risk [T1-2, N0 and Nx, M0 and Mx], low-to-intermediate risk [T3, N0 and Nx, M0 and Mx; T1-3, N1, M0 and Mx] and high-risk [T4, any N, any M; M1, any T, any N]), RAI therapy. Outcome of interest was cancer-specific survival (CSS), defined as the date of diagnosis to the date of death from thyroid cancer.

### 2.3 Study analysis

The data extracted from SEER program were analyzed by SPSS statistical software, version 26.0 (IBM Corp, Armonk, NY). The differences between the case group (RAI) and the control group (no-RAI) were compared using *x^2^* test or Fisher’s exact tests. Multivariate COX regression models were performed to estimate prognostic factors for CSS. Hazard ratios (HRs) and 95% confidence index (CI) were displayed. P values less than 0.05 were considered statistically significant. CSS curves were evaluated according to Kaplan-Meier analysis and log-rank test. PSM was performed to reduce the differences in patient baseline characteristics and selection bias. In the cohort, age, sex, race, T stage, N stage, and M stage were included as covariates for PSM. PTC, OCA, and FTC patients were performed 1:1 nearest neighbor matching with a matching tolerance of 0.002, 0.005, and 0.001, respectively, to obtain matched pairs.

## 3 Results

### 3.1 Patient characteristics

**Table 1** showed patients characteristics in our study and 105195 DTC patients (including 98288 PTC, 2153 OCA and 4754 FTC) receiving TT were finally included in our cohort. The differences in the demographic and clinical characteristics between case group (RAI) and control group (no-RAI) were exhibited in **Table 2**. In three types of DTC, patients undergoing RAI had higher T stage, M stage, and ATA risk staging compared with patients without. PTC (P<0.001) and FTC (P=0.039) patients receiving RAI exhibited higher N stage compared to those without, but not OCA (P=0.237). **Supplementary Table 1** displayed the differences in the clinicopathological features and treatment of the three pathological types. The rates of RAI therapy in PTC, OCA, and FTC were 52.3%, 64.4%, and 63.8% (P<0.001), respectively. The PSM cohort was presented in **Supplementary Table 2** and all P value > 0.05.

**Table 1:** Patient characteristics in DTC ^a^.

Variables	n=105195
Follow-up time	
median (range), months	86 (1-191)
Age	
<55	65975 (62.7)
≥55	39220 (37.3)
Sex	
Female	80867 (76.9)
Male	24328 (23.1)
Race	
White	85646 (81.4
Black	6261 (6)
Other	12178 (11.6)
Unknown	1110 (1.1)
Pathology	
PTC^b^	98288 (93.4)
OCA^c^	2153 (2)
FTC^d^	4754 (4.5)
T stage	
T1	60001 (57)
T2	18477 (17.6)
T3	23228 (22.1)
T4	3489 (3.3)
N stage	
N0 and Nx	78442 (74.6)
N1	26753 (25.4)
M stage	
M0 and Mx	104192 (99)
M1	1003 (1)
ATA^e^ risk staging (TNM)	
low risk	63232 (60.1)
low to intermediate risk	37762 (35.9)
high risk	4201 (4)
RAI^f^	
Yes	55854 (53.1)
No	49341 (46.9)

^a^DTC, differentiated thyroid carcinoma.

^b^PTC, papillary thyroid carcinoma.

^c^OCA, oncocytic carcinoma of thyroid.

^d^FTC, Follicular thyroid carcinoma.

^e^ATA, American Thyroid Association.

^f^RAI, Radioactive iodine.

**Table 2:** Patients Characteristics in PTC^g^ , OCA^h^ , and FTC^i^.

Variables	PTC			OCA			FTC		
	RAI^j^	non-RAI	P	RAI	non-RAI	P	RAI	non-RAI	P
	n=51435	n=46853		n=1387	n766		n=3032	n=1722	
Age									
<55	34275 (66.6)	28094 (60.0)	<0.001	625 (45.1)	307 (40.1)	0.025	1699 (56.0)	975 (56.6)	0.696
≥55	17160 (33.4)	18759 (40.0)		762 (54.9)	459 (59.9)		1333 (44.0)	747 (43.4)	
Sex									
Female	38383 (74.6)	37621 (80.3)	<0.001	926 (66.8)	543 (70.9)	0.049	2108 (69.5)	1286 (74.7)	<0.001
Male	13052 (25.4)	9232 (19.7)		461 (33.2)	223 (29.1)		924 (30.5)	436 (25.3)	
Race									
White	41928 (81.5)	38170 (81.5)	<0.001	1178 (84.9)	657 (85.8)	0.424	2371 (78.2)	1342 (77.9)	0.011
Black	2405 (4.7)	3147 (6.7)		100 (7.2)	44 (5.7)		363 (12.0)	202 (11.7)	
Other	6707 (13.0)	4879 (10.4)		99 (7.1)	56 (7.3)		283 (9.3)	154 (8.9)	
Unknown	395 (0.8)	657 (1.4)		10 (0.7)	9 (1.2)		15 (0.5)	24 (1.4)	
T stage									
T1	23249 (45.2)	35084 (74.9)	<0.001	306 (22.1)	237 (30.9)	<0.001	596 (19.7)	529 (30.7)	<0.001
T2	10359 (20.1)	5437 (11.6)		502 (36.2)	273 (35.6)		1241 (40.9)	665 (38.6)	
T3	15352 (29.8)	5498 (11.7)		530 (38.2)	225 (29.4)		1127 (37.2)	496 (28.8)	
T4	2475 (4.8)	834 (1.8)		49 (3.5)	31 (4.0)		68 (2.2)	32 (1.9)	
N stage									
N0 and Nx	31419 (61.1)	40398 (86.2)	<0.001	1297 (93.5)	726 (94.8)	0.237	2923 (96.4)	1679 (97.5)	0.039
N1	20016 (38.9)	6455 (13.8)		90 (6.5)	40 (5.2)		109 (3.6)	43 (2.5)	
M stage									
M0 and Mx	50805 (98.8)	46662 (99.6)	<0.001	1353 (97.5)	757 (98.8)	0.043	2918 (96.2)	1697 (98.5)	<0.001
M1	630 (1.2)	191 (0.4)		34 (2.5)	9 (1.2)		114 (3.8)	25 (1.5)	
ATA^k^ risk staging (TNM)									
Low risk	22791 (44.30)	36251 (77.40)	<0.001	768 (55.4)	492 (64.2)	<0.001	1758 (58.0)	1172 (68.1)	<0.001
Low to intermediate risk	25717 (50.0)	9654 (20.6)		545 (39.3)	237 (30.9)		1107 (36.5)	502 (29.2)	
High risk	2927 (5.7)	948 (2.0)		74 (5.3)	37 (4.8)		167 (5.5)	48 (2.8)	

^g^PTC, papillary thyroid carcinoma.

^h^OCA, oncocytic carcinoma of thyroid.

^i^FTC, Follicular thyroid carcinoma.

^j^RAI, Radioactive iodine.

^k^ATA, American Thyroid Association.

### 3.2 Survival analysis and prognostic factors

Univariate analysis in distinct types of DTC was presented in **Supplementary Table 3**. **Table 3** showed the prognostic factors for CSS in three types of DTC by multivariate analyses before and after PSM. The median follow-up time in PTC, OCA, and FTC was 86 months (range from 1 to 191), 94 months (range from 1 to 191), and 91 months (range from 1 to 191), respectively. **Table 4** exhibited 5-years, 10-years, and 15-years CSS rate for DTC according to pathological types in the entire cohort. PTC had the best CSS rate, FTC the second and OCA the worst (P<0.001). The prognostic factors associated with CSS in PTC included age, sex, and T stage, and N stage, and M stage, and RAI in entire cohort and PSM cohort. With regard to OCA and FTC, age, T stage, and N stage, and M stage were independent risk factors related to CSS in entire cohort and PSM cohort. Inconsistent with PTC, RAI was no prognostic factor after PSM whether in OCA (Yes as reference, HRs=1.128; 95% CI=0.699-1.82; P=0.622) or FTC (Yes as reference, HRs=1.492; 95% CI=0.958-2.325; P=0.077).

**Table 3:** Prognostic factors in PTC^l^ , OCA^m^ , and FTC^n^ patients.

Variables		Before PSM^o^			After PSM		
		HRs^p^	95.0% CI^q^	P	HRs	95.0% CI	P
PTC							
Age	<55	1	Reference		1	Reference	
	≥55	7.23	6.295-8.304	<0.001	7.519	6.243-9.056	<0.001
Sex	Female	1	Reference		1	Reference	
	Male	1.599	1.429-1.789	<0.001	1.563	1.347-1.814	<0.001
Race	White	1	Reference		1	Reference	
	Black	1.119	0.876-1.429	0.368	1.056	0.771-1.447	0.735
	Other	0.995	0.849-1.166	0.948	0.828	0.668-1.028	0.087
T stage	T1	1	Reference		1	Reference	
	T2	2.035	1.652-2.506	<0.001	1.925	1.486-2.493	<0.001
	T3	4.158	3.547-4.874	<0.001	4.042	3.295-4.959	<0.001
	T4	12.829	10.778-15.271	<0.001	13.688	10.96-17.094	<0.001
N stage	N0 and Nx	1	Reference		1	Reference	
	N1	2.036	1.801-2.301	<0.001	2.155	1.838-2.527	<0.001
M stage	M0 and Mx	1	Reference		1	Reference	
	M1	6.855	5.855-8.026	<0.001	7.403	5.996-9.139	<0.001
RAI^r^	Yes	1	Reference		1	Reference	
	No	1.213	1.077-1.367	0.002	1.154	1-1.332	0.05
OCA							
Age	<55	1	Reference		1	Reference	
	≥55	3.043	1.805-5.13	<0.001	4.837	2.186-10.705	<0.001
Sex	Female	1	Reference		1	Reference	
	Male	1.001	0.664-1.51	0.995	0.996	0.596-1.666	0.989
Race	White	1	Reference		1	Reference	
	Black	0.685	0.276-1.699	0.414	0.766	0.276-2.127	0.608
	Other	1.23	0.63-2.401	0.544	1.102	0.461-2.629	0.828
T stage	T1	1	Reference		1	Reference	
	T2	1.455	0.591-3.578	0.414	1.286	0.466-3.548	0.627
	T3	5.411	2.436-12.021	<0.001	5.534	2.307-13.275	<0.001
	T4	10.488	4.158-26.455	<0.001	10.015	3.523-28.475	<0.001
N stage	N0 and Nx	1	Reference		1	Reference	
	N1	2.242	1.264-3.979	0.006	2.882	1.423-5.834	0.003
M stage	M0 and Mx	1	Reference		1	Reference	
	M1	8.353	4.612-15.126	<0.001	3.948	1.65-9.447	0.002
RAI	Yes	1	Reference		1	Reference	
	No	1.27	0.836-1.93	0.262	1.128	0.699-1.82	0.622
FTC							
Age	<55	1	Reference		1	Reference	
	≥55	3.644	2.373-5.594	<0.001	4.477	2.576-7.779	<0.001
Sex	Female	1	Reference		1	Reference	
	Male	0.836	0.587-1.192	0.322	0.844	0.513-1.386	0.502
Race	White	1	Reference		1	Reference	
	Black	0.974	0.592-1.604	0.918	0.916	0.448-1.873	0.811
	Other	1.216	0.767-1.93	0.405	1.455	0.812-2.606	0.207
T stage	T1	1	Reference		1	Reference	
	T2	2.399	1.101-5.227	0.028	2.007	0.887-4.541	0.095
	T3	6.185	2.956-12.941	<0.001	4.773	2.209-10.316	<0.001
	T4	14.542	6.36-33.247	<0.001	12.868	4.791-34.562	<0.001
N stage	N0 and Nx	1	Reference		1	Reference	
	N1	3.442	2.242-5.283	<0.001	3.679	1.794-7.543	<0.001
M stage	M0 and Mx	1	Reference		1	Reference	
	M1	9.502	6.359-14.199	<0.001	7.476	3.798-14.713	<0.001
RAI	Yes	1	Reference		1	Reference	
	No	1.589	1.108-2.278	0.012	1.492	0.958-2.325	0.077

^l^PTC, papillary thyroid carcinoma.

^m^OCA, oncocytic carcinoma of thyroid.

^n^FTC, follicular thyroid carcinoma.

^o^PSM, propensity score–matched.

^p^HRs, Hazard ratios.

^q^CI, confidence index.

^r^RAI, Radioactive iodine.

**Table 4:** CSS^s^ comparison in PTC^t^ , FTC^u^, and OCA^v^ patients.

	PTC	FTC	OCA	P
5-years CSS (%)	99.2	97.9	97.1	<0.001
10-years CSS (%)	98.2	96.0	94.1	<0.001
15-years CSS (%)	97.3	94.9	91.6	<0.001

^s^CSS, cancer-specific survival.

^t^PTC, papillary thyroid carcinoma.

^u^OCA, oncocytic carcinoma of thyroid.

^v^FTC, Follicular thyroid carcinoma.

### 3.3 The benefits of RAI in different types of DTC are vary

#### 3.3.1 The benefit of RAI in LNM or T4 stage or DM

**Figure 1:**
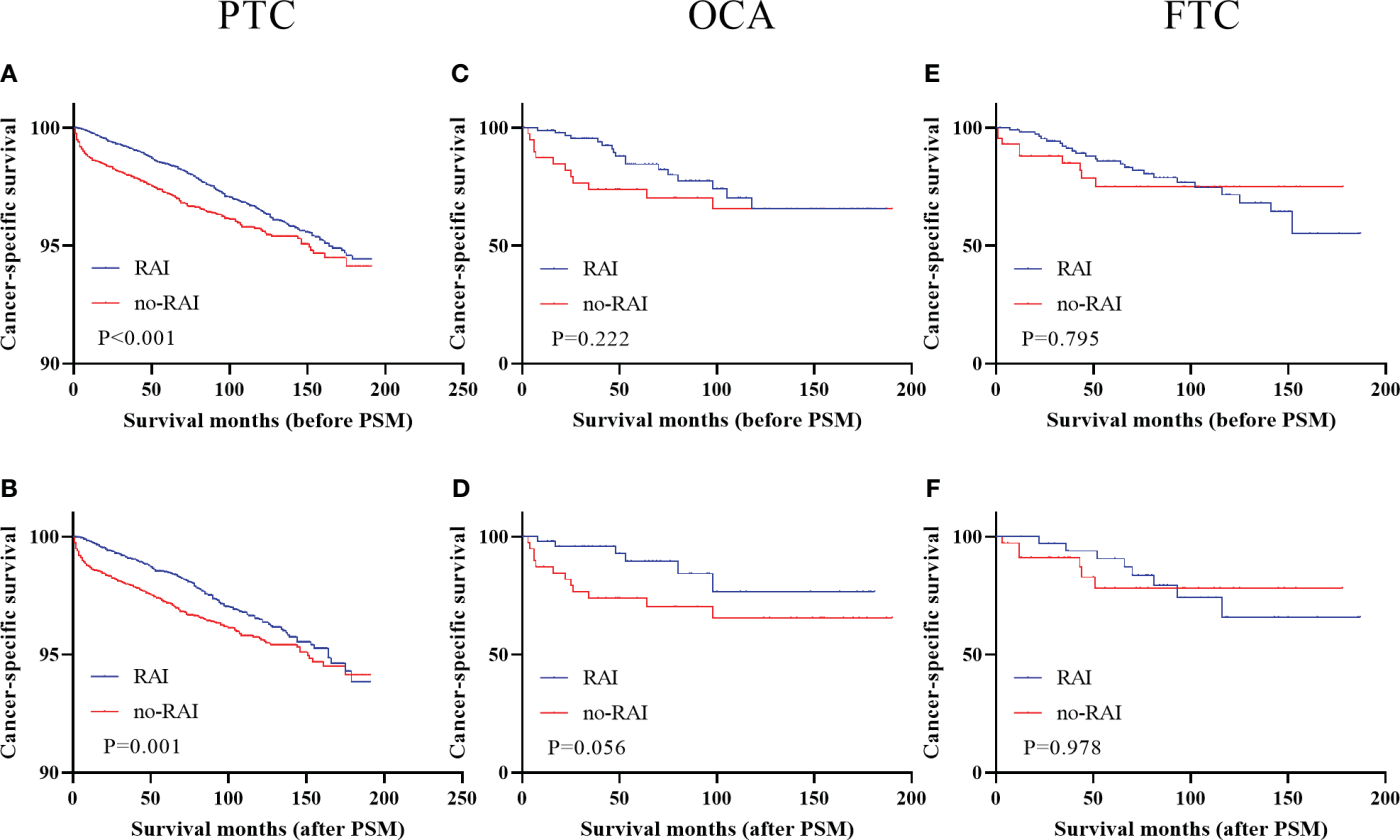
Cancer-specific survival for distinct types of DTC with lymph node metastases in the entire cohort and PSM cohort **(A)** PTC before PSM **(B)** PTC after PSM **(C)** OCA before PSM **(D)** OCA after PSM **(E)** FTC before PSM **(F)** FTC after PSM. DTC, differentiated thyroid carcinoma; PSM, propensity score–matched; PTC, papillary thyroid carcinoma; OCA, oncocytic carcinoma of thyroid; FTC, Follicular thyroid carcinoma; RAI, Radioactive iodine.

Kaplan-Meier curves were performed to analyze the effect of RAI in three types of DTC when LNM or gross extrathyroidal extension (T4 stage) or DM were presented. Survival analysis of three types of DTC with LNM were showed in **Figure 1** (**Figure 1**). Only PTC patients with LNM could improving CSS when receiving RAI in entire cohort (**Figure 1A**, P<0.001) and PSM cohort (**Figure 1B**, P=0.001). RAI could not significantly improve CSS of OCA and FTC patients when LNM were presented either in entire cohort (OCA: **Figure 1C**, P=0.222; FTC: **Figure 1E**, P=0.795) or PSM cohort (OCA: **Figure 1D**, P=0.056; FTC: **Figure 1F**, P=0.978).

**Figure 2:**
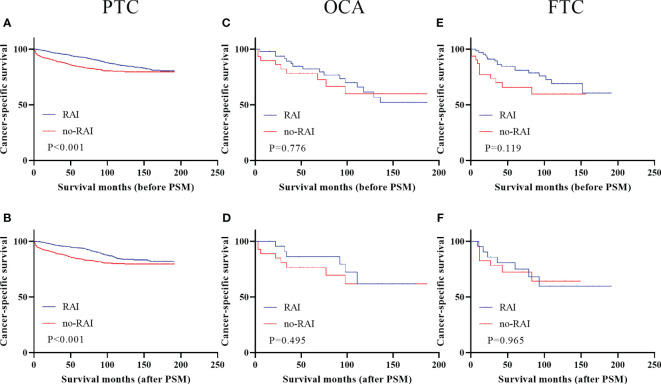
Cancer-specific survival for distinct types of DTC with T4 stage in the entire cohort and PSM cohort **(A)** PTC before PSM **(B)** PTC after PSM **(C)** OCA before PSM **(D)** OCA after PSM **(E)** FTC before PSM **(F)** FTC after PSM. DTC, differentiated thyroid carcinoma; PSM, propensity score–matched; PTC, papillary thyroid carcinoma; OCA, oncocytic carcinoma of thyroid; FTC, Follicular thyroid carcinoma; RAI, Radioactive iodine.

**Figure 2** showed the survival curves of DTC patients with T4 stage before and after PSM. Only PTC patients with T4 stage had better survival outcomes when receiving RAI in entire cohort (**Figure 2A**, P<0.001) and PSM cohort (**Figure 2B**, P<0.001). OCA and FTC had no significant improvements when undergoing RAI (entire cohort: OCA, **Figure 2C**, P=0.776; FTC, **Figure 2E**, P=0.119; PSM cohort: OCA, **Figure 2D**, P=0.495; FTC, **Figure 2F**, P=0.965).

**Figure 3:**
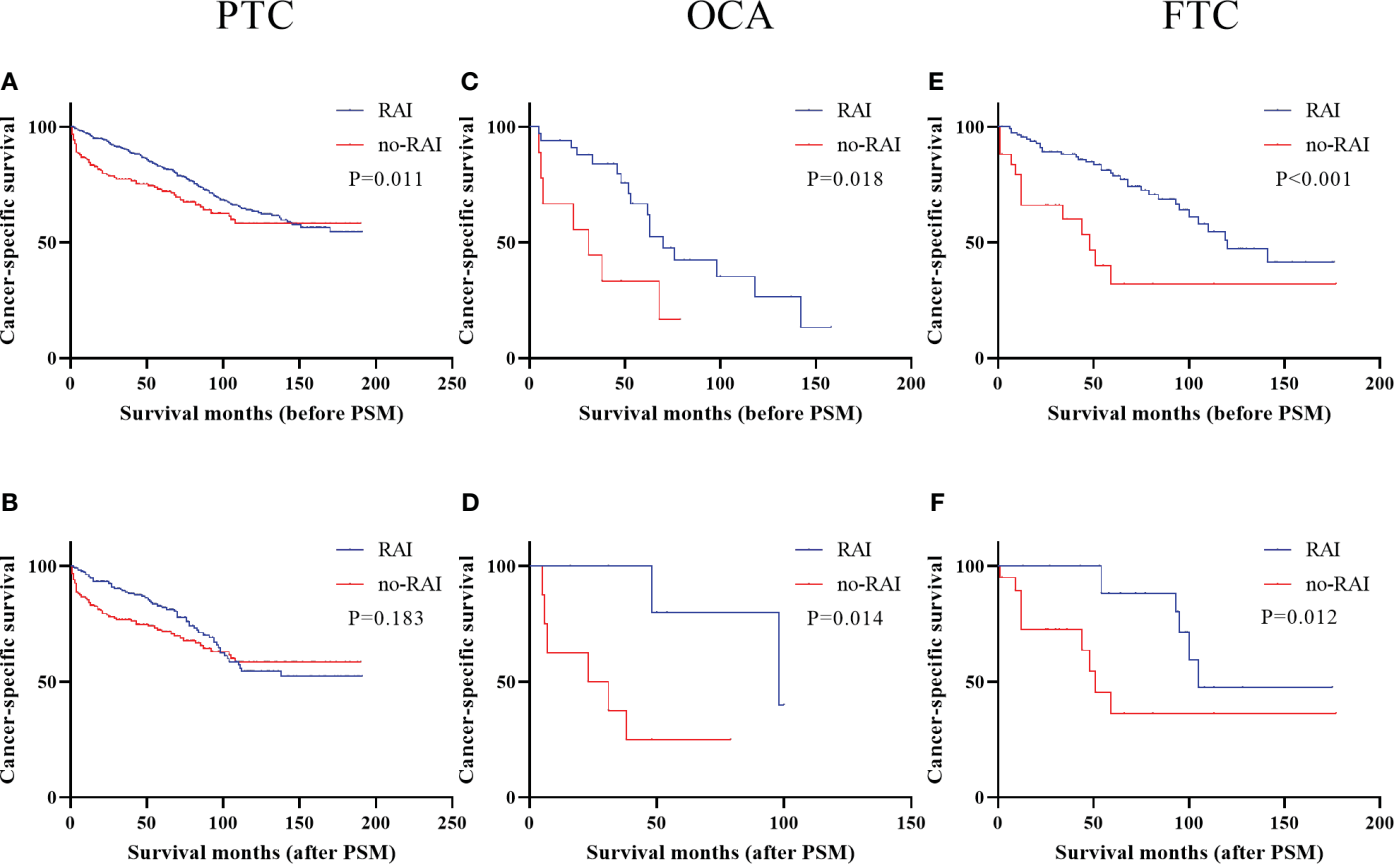
Cancer-specific survival for distinct types of DTC with distant metastases in the entire cohort and PSM cohort **(A)** PTC before PSM **(B)** PTC after PSM **(C)** OCA before PSM **(D)** OCA after PSM **(E)** FTC before PSM **(F)** FTC after PSM. DTC, differentiated thyroid carcinoma; PSM, propensity score–matched; PTC, papillary thyroid carcinoma; OCA, oncocytic carcinoma of thyroid; FTC, Follicular thyroid carcinoma; RAI, Radioactive iodine.

Kaplan-Meier curves of DTC patients with DM were presented in **Figure 3**. Metastatic OCA and FTC patients could benefit from RAI in entire cohort (OCA: **Figure 3C**, P=0.018; FTC: **Figure 3D**, P<0.001) and PSM cohort (OCA: **Figure 3E**, P=0.014; FTC: **Figure 3F**, P=0.012). Metastatic PTC patients had better survival outcomes in entire cohort (**Figure 3A**, P<0.001), but not in PSM cohort (**Figure 3B**, P=0.183). We further analyzed the 50 months, 100 months and 150 months CSS in PTC patients with DM (**Table 5**). In entire cohort, RAI therapy improved the 50 months (P<0.001), 100 months (P=0.003) and 150 months (P=0.008) CSS of metastatic PTC patients. In PSM cohort, Patients receiving RAI only showed significant differences in 50 months CSS.

**Table 5:** 50 months, 100 months, 150 months CSS^w^ analysis in metastatic PTC^x^ patients.

	Before PSM^y^				After PSM			
Follow-up time	0 months	50 months	100 months	150 months	0 months	50 months	100 months	150 months
RAI^z^	630	389	171	48	214	131	52	17
No-RAI	191	103	44	13	187	100	44	13
P		< 0.001	0.003	0.008		0.001	0.09	0.183
Accumulated deaths	0	125	196	216	0	71	109	119
Number at risk	821	492	215	61	401	231	96	30

^w^CSS, cancer-specific survival.

^x^PTC, papillary thyroid carcinoma.

^y^PSM, propensity score–matched.

^z^RAI, Radioactive iodine.

#### 3.3.2 The benefit of RAI according to ATA risk staging (TNM)

**Figure 4:**
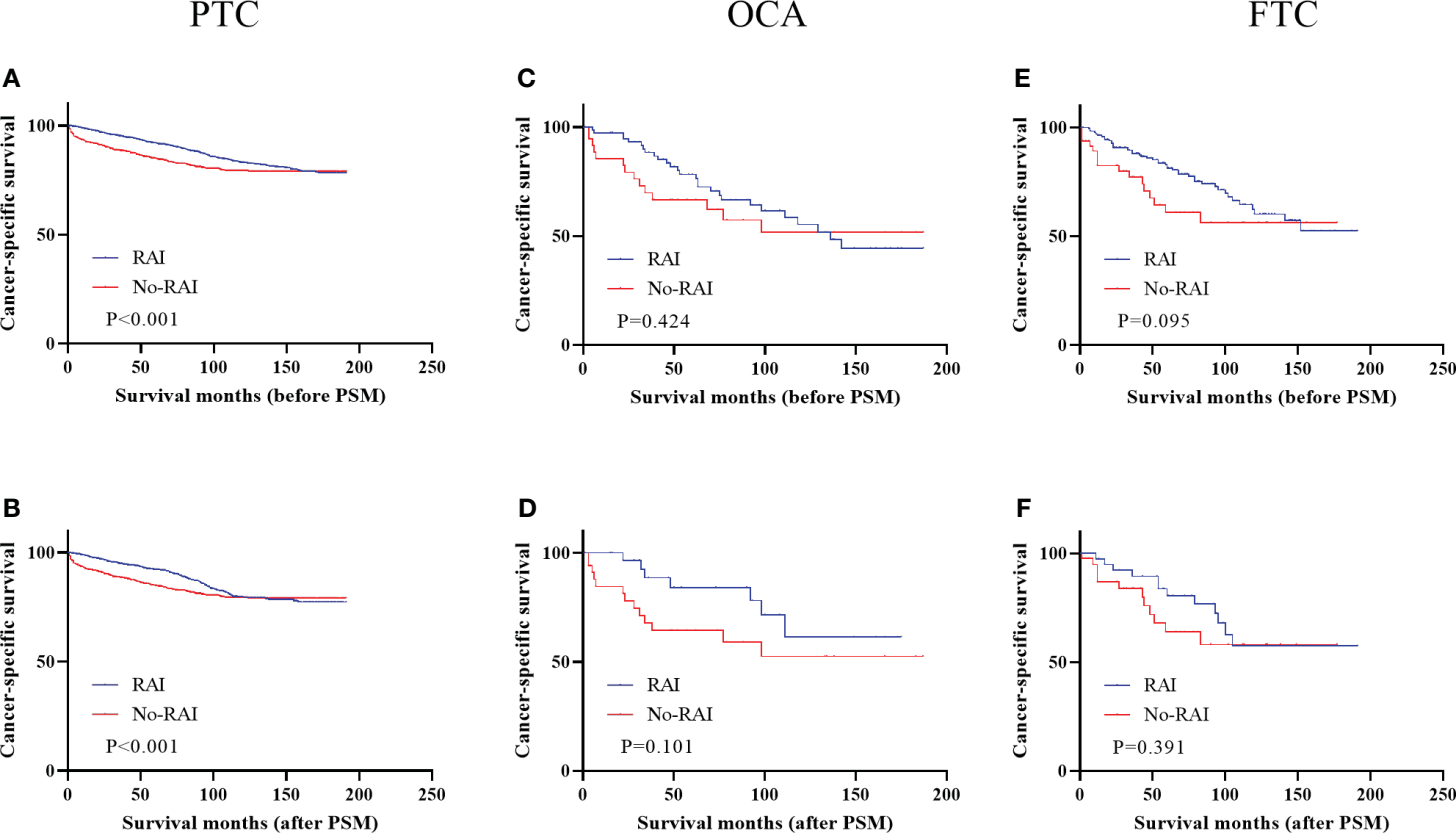
Cancer-specific survival for distinct types of DTC with high-risk staging in the entire cohort and PSM cohort **(A)** PTC before PSM **(B)** PTC after PSM **(C)** OCA before PSM **(D)** OCA after PSM **(E)** FTC before PSM **(F)** FTC after PSM. DTC, differentiated thyroid carcinoma; PSM, propensity score–matched; PTC, papillary thyroid carcinoma; OCA, oncocytic carcinoma of thyroid; FTC, Follicular thyroid carcinoma; RAI, Radioactive iodine.

**Figure 4** showed the effect of RAI in high-risk DTC patients. Only PTC patients had better CSS when receiving RAI in entire cohort (**Figure 4A**, P<0.001) and PSM cohort (**Figure 4B**, P<0.001). In OCA and FTC patients, RAI had no significant improvement in CSS whether in entire cohort (OCA: **Figure 4C**, P=0.424; FTC: **Figure 4E**, P=0.101) or PSM cohort (OCA: **Figure 4D**, P=0.095; FTC: **Figure 4F**, P=0.391). Survival analysis of low-risk patients and low-to-intermediate-risk patients were displayed in **Supplementary Figure 1** before and after PSM and there were no significant improvements in all three types of DTC.

## 4 Discussion

TC was a relatively indolent cancer with a high 5-year survival rate of 98%, but it still affected a significant number of individuals each year, with an incidence rate of 14.6 per 100,000 men and women (25, 26). While thyroidectomy was the main treatment for DTC, postoperative therapies, including thyroid stimulating hormone suppression therapy, RAI therapy and tyrosine kinase inhibitors (TKIs) therapy, were also available (27). TKIs were primarily used for iodine-refractory TC and currently tested in clinical trials (28). However, RAI therapy remained a primary treatment for high-risk DTC after TT, aside from drug therapy. Despite its effectiveness, RAI therapy could have adverse effects, including dysfunction of salivary gland and lacrimal gland, testicular dysfunction, transient female gonadal dysfunction and second primary malignancies (15, 29, 30). Inappropriate RAI therapy could have an impact on quality of life and shorten survival time. Therefore, we aimed to refine RAI therapy strategies for DTCs in clinical practice, balancing the complications of RAI therapy.

Previous studies found that OCA and FTC had a worse prognosis compared with PTC (12, 13). Our study was consistent with those findings, revealing that PTC had the best prognosis, followed by FTC, with OCA having the worst prognosis. The 10-year CSS rates for PTC, FTC, and OCA were 98.2%, 96%, and 94.1%, respectively. We considered that three types of DTC patients differed in RAI response. RAI therapy was an independent risk factor associated with CSS in PTC but not OCA and FTC. Specifically, In the presence of LNM or gross extrathyroidal extension, PTC patients could benefit from RAI therapy, while such benefit was not observed in OCA and FTC patients. Additionally, in OCA and FTC patients with DM, RAI therapy improved CSS. There are differences in survival analysis results of metastatic PTC patients before and after PSM. The conclusion that RAI treatment could improve early survival (50 months CSS) of patients with metastatic PTC remains consistent. However, statistical differences were observed in the analysis of 100 months and 150 months CSS in the entire cohort, but not in the PSM cohort. It was found that the sample size of patients with long-term follow-up (100 months and 150 months) in the PSM cohort was limited. Additionally, a significant amount of data for the RAI group were deleted in the PSM cohort, and even non-RAI group data were not screened in the long-term follow-up analysis, which could compromise the test efficiency. Therefore, a larger sample size is required to support the analysis of long-term survival in patients with metastatic PTC, especially in the context of RAI treatment. We further compared the differences in RAI response among DTC according to ATA risk staging (TNM). In high-risk patients, RAI therapy could improve prognosis in PTC but not in OCA and FTC. In low-risk and low-to-intermediate-risk patients, RAI therapy did not provide any benefit. Our study suggested that RAI therapy was not recommended for OCA and FTC patients with regional lesions only.

Our study was consistent with previous research, indicating that OCA and FTC could benefit from RAI. Chow et al. considered that RAI therapy could improve survival outcomes and reduce recurrence and was an effective treatment for FTC (21). A SEER-based research concluded that metastatic FTC with receiving RAI had a better overall survival than those who did not (23). A retrospective study of 251 patients showed that RAI therapy was unnecessary in minimally invasive FTC and did not improve distant-metastases-free survival or CSS (20). This conclusion about minimally invasive FTC was not contradictory to our findings that regional lesions could not benefit from RAI therapy. Jillard et al. similarly considered that RAI therapy was associated with a better prognosis in OCA with advanced TNM stage (24).

The effectiveness of RAI therapy in the three types of DTC cannot be completely equated. Our finding revealed that fewer patients could benefit from RAI therapy for OCA and FTC compared to PTC. However, the percentage of patients receiving RAI therapy in our sample was higher in OCA (64.4%) and FTC (63.8%) than in PTC (52.3%). This might be due to the more aggressive clinicopathological features and biological behaviors and poorer prognosis of OCA and FTC compared to PTC, which might lead clinicians to prefer more aggressive treatment strategies for OCA and FTC. Previous research had shown differences in RAI avidity and RAI response among the three types of DTC (16). OCA (13.6%) had the lowest RAI avidity, classical variant PTC (21.4%) had medium RAI avidity, and FTC (76.5%) and follicular variant PTC (75.6%) had highest RAI avidity. However, compared to classical and follicular variant PTC, OCA and FTC had lower RAI response and more disease progression after RAI therapy. Chindris et al. found that only 2 of 27 OCA with lung metastases showed positive RAI scans (31). A retrospective study of metastatic PTC and FTC concluded that 137 of 178 PTC patients and 156 of 245 FTC patients observed RAI avidity and 41 PTC patients and 29 FTC patients achieved remission after RAI therapy (32). The differences in pathology and genomics of PTC, OCA and FTC might contribute to the differences in RAI avidity among them. Classical variant PTC, the most common type of PTC, was characterized by papillae and nuclear changes with BRAF^V600E^ mutation being the most frequent mutation. This mutation reduced expression of functional thyroid genes including those encoding thyroglobulin and sodium/iodide symporter (NIS) proteins through high mitogen-activated protein kinase (MAPK) pathway output, resulting in a decrease in RAI activity (33, 34). FTC was associated with mutually exclusive mutations in the RAS or PAX8-PPARG fusion oncogenes and lacked the BRAF^V600E^ mutation, which might lead to higher RAI avidity compared to conventional PTC (35). However, in OCA, which was characterized by a high rate of mitochondrial mutations and amplifications of BRAF, rather than activating mutations were observed (36). TERT promoter mutations were observed in 22% OCA and Liu et al. found that TERT mutations were associated with loss of RAI avidity (36, 37). Current researches had yet to fully explain the low RAI avidity observed in OCA. There were pitfalls of RAI therapy that not all RAI uptake resulted in RAI response, which could be observed in poorly differentiated tumors, in older patients or in patients with a heavy tumor burden (38). One possible explanation for this phenomenon was that the heterogeneity within the tumor which could result in abnormal distribution of RAI that only killed functional tumor cells and left non-iodine taking cells (27).

Our study highlighted the importance of distinguishing the response to RAI therapy among the three types of DTC and individualizing treatment strategies accordingly. Further researches were needed to investigate the mechanisms and molecular markers associated with response to RAI treatment.

Our research had several limitations. Firstly, due to its retrospective study, there was the possibility of selection bias. Secondly, The SEER database lacked data on disease recurrence and secondary surgery, so we were unable to analyze the disease-free survival rate after RAI treatment.

## 5 Conclusion

RAI therapy was an effective treatment for DTC and should be considered individually in PTC, OCA and FTC patients. OCA and FTC patients with DM rather than with regional lesions only could benefit from RAI therapy. However, with regard to PTC, RAI therapy could improve survival outcomes when gross extrathyroidal extension or LNM and early survival when DM were presented. Our results provided further guideline for treatment selection in DTC.

## Data availability statement

The raw data supporting the conclusions of this article will be made available by the authors, without undue reservation.

## Ethics statement

The studies involving human participants were reviewed and approved by Union Hospital, Tongji Medical College, Huazhong University of Science and Technology. The patients/participants provided their written informed consent to participate in this study.

## Author contributions

HHG: Conceptualization, Methodology, Data curation, Formal analysis, Figures and Tables creation, Writing - Original Draft, Writing - Review and Editing. NS and TH: Conceptualization, Methodology, Formal analysis, Writing - Review and Editing. NZ: Data curation, Formal analysis, Figures and Tables creation, Writing - Review and Editing. YXH and FRZ: Formal analysis, Figures and Tables creation. All authors contributed to the article and approved the submitted version.

## Supplementary material

Supplementary Figure 1 Cancer-specific survival for distinct types of DTC with low-risk or low-to-intermediate-risk staging in the entire cohort and PSM cohort. Low-risk DTC: **(A)** PTC before PSM **(B)** PTC after PSM **(E)** OCA before PSM **(F)** OCA after PSM **(I)** FTC before PSM **(J)** FTC after PSM. Low-to-intermediate-risk staging: **(C)** PTC before PSM **(D)** PTC after PSM **(J)** OCA before PSM **(H)** OCA after PSM **(K)** FTC before PSM **(L)** FTC after PSM. DTC, differentiated thyroid carcinoma; PSM, propensity score–matched; PTC, papillary thyroid carcinoma; OCA, oncocytic carcinoma of thyroid; FTC, Follicular thyroid carcinoma; RAI, Radioactive iodine.
