# Supplementary material for: Radioactive iodine therapy strategies for distinct types of differentiated thyroid cancer: a propensity score–matched analysis
Source: Front Endocrinol (Lausanne). 2023 Aug 17;14:1158581. doi: 10.3389/fendo.2023.1158581 (PMC10471126; doi:10.3389/fendo.2023.1158581)
Supplement: Supplementary file 1 [file Table_1.docx]

| **Supplementary Table. 1**Patients Characteristics comparison in PTC, OCA, and FTC | | | | |
| --- | --- | --- | --- | --- |
| Variables | PTC | HCC | FTC | P |
|  | n=98288 | n=2153 | n=4754 |  |
| Age |  |  |  |  |
| <55 | 62369(63.5) | 932(43.3) | 2674(56.2) | <0.001 |
| ≥55 | 35919(36.5) | 1221(56.7) | 2080(43.8) |  |
| Sex |  |  |  |  |
| Female | 76004(77.3) | 1469(68.2) | 3394(71.4) | <0.001 |
| Male | 22284(22.7) | 684(31.8) | 1360(28.6) |  |
| Race |  |  |  |  |
| White | 80098(81.5) | 1835(85.2) | 3713(78.1) | <0.001 |
| Black | 5552(5.6) | 144(6.7) | 565(11.9) |  |
| Other | 11586(11.8) | 155(7.2) | 437(9.2) |  |
| Unknown | 1052(1.1) | 19(0.9) | 39(0.8) |  |
| T stage |  |  |  |  |
| T1 | 58333(59.3) | 543(25.2) | 1125(23.7) | <0.001 |
| T2 | 15796(16.1) | 775(36.0) | 1906(40.1) |  |
| T3 | 20850(21.2) | 755(35.1) | 1623(34.1) |  |
| T4 | 3309(3.4) | 80(3.7) | 100(2.1) |  |
| N stage |  |  |  |  |
| N0andNx | 71817(73.1) | 2023(94.0) | 4602(96.8) | <0.001 |
| N1 | 26471(26.9) | 130(6.0) | 152(3.2) |  |
| M stage |  |  |  |  |
| M0andMx | 97467(99.2) | 2110(98.0) | 4615(97.1) | <0.001 |
| M1 | 821(0.8) | 43(2) | 139(2.9) |  |
| ATA Risk staging (TNM) |  |  |  |  |
| low risk | 59042(60.1) | 1260(58.5) | 2930(61.6) | 0.001 |
| low to intermediate risk | 35371(36.0) | 782(36.3) | 1609(33.8) |  |
| high risk | 3875(3.9) | 111(5.2) | 215(4.5) |  |
| RAI |  |  |  |  |
| Yes | 51435(52.3) | 1387(64.4) | 3032(63.8) | <0.001 |
| No | 46853(47.7) | 766(35.6) | 1722(36.2) |  |
| Abbreviations: PTC=papillary thyroid carcinoma; OCA=oncocytic carcinoma of thyroid; FTC=Follicular thyroid carcinoma; RAI=Radioactive iodine; ATA=American Thyroid Association | | | | |
