# Supplementary material for: Radioactive iodine therapy strategies for distinct types of differentiated thyroid cancer: a propensity score–matched analysis
Source: Front Endocrinol (Lausanne). 2023 Aug 17;14:1158581. doi: 10.3389/fendo.2023.1158581 (PMC10471126; doi:10.3389/fendo.2023.1158581)
Supplement: Supplementary file 2 [file Table_2.docx]

| **Supplementary Table. 2** Patients Characteristics in PTC, OCA, and FTC patients in PSM cohort | | | | | | | | | |
| --- | --- | --- | --- | --- | --- | --- | --- | --- | --- |
| Variables | PTC | | | OCA | | | FTC | | |
|  | RAI | non-RAI | P | RAI | non-RAI | P | RAI | non-RAI | P |
|  | n=30483 | n=30483 |  | n=751 | n=751 |  | n=1674 | n=1674 |  |
| Age |  |  |  |  |  |  |  |  |  |
| <55 | 19851(65.1) | 19838(65.1) | 0.912 | 294(39.1) | 306(40.7) | 0.527 | 932(55.7) | 957(57.2) | 0.384 |
| ≥55 | 10632(34.9) | 10645(34.9) |  | 457(60.9) | 445(59.3) |  | 742(44.3) | 717(42.8) |  |
| Sex |  |  |  |  |  |  |  |  |  |
| Female | 23759(77.9) | 23818(78.1) | 0.564 | 530(70.6) | 531(70.7) | 0.955 | 1271(75.9) | 1247(74.5) | 0.337 |
| Male | 6724(22.1) | 6665(21.9) |  | 221(29.4) | 220(29.3) |  | 403(24.1) | 427(25.5) |  |
| Race |  |  |  |  |  |  |  |  |  |
| White | 24832(81.5) | 24825(81.4) | 0.76 | 637(84.8) | 649(86.4) | 0.514 | 1290(77.1) | 1313(78.4) | 0.648 |
| Black | 1631(5.4) | 1670(5.5) |  | 52(6.9) | 40(5.3) |  | 223(13.3) | 200(11.9) |  |
| Other | 3706(12.2) | 3658(12.0) |  | 52(6.9) | 55(7.3) |  | 149(8.9) | 151(9.0) |  |
| Unknown | 314(1.0) | 330(1.1) |  | 10(1.3) | 7(0.9) |  | 12(0.7) | 10(0.6) |  |
| T stage |  |  |  |  |  |  |  |  |  |
| T1 | 18725(61.4) | 18725(61.4) | 0.983 | 206(27.4) | 225(30.0) | 0.574 | 491(29.3) | 497(29.7) | 0.776 |
| T2 | 5432(17.8) | 5427(17.8) |  | 294(39.1) | 272(36.2) |  | 645(38.5) | 659(39.4) |  |
| T3 | 5508(18.1) | 5497(18.0) |  | 226(30.1) | 225(30.0) |  | 517(30.9) | 493(29.5) |  |
| T4 | 818(2.7) | 834(2.7) |  | 25(3.3) | 29(3.9) |  | 21(1.3) | 25(1.5) |  |
| N stage |  |  |  |  |  |  |  |  |  |
| N0 and Nx | 24046(78.9) | 24044(78.9) | 0.984 | 701(93.3) | 711(94.7) | 0.277 | 1639(97.9) | 1638(97.8) | 0.905 |
| N1 | 6437(21.1) | 6439(21.1) |  | 50(6.7) | 40(5.3) |  | 35(2.1) | 36(2.2) |  |
| M stage |  |  |  |  |  |  |  |  |  |
| M0 and Mx | 30269(99.3) | 30296(99.4) | 0.176 | 743(98.9) | 743(98.9) | 1 | 1653(98.7) | 1654(98.8) | 0.875 |
| M1 | 214(0.7) | 187(0.6) |  | 8(1.10) | 8(1.10) |  | 21(1.3) | 20(1.2) |  |
| ATA Risk staging (TNM) |  |  |  |  |  |  |  |  |  |
| low risk | 20077(65.9) | 19900(65.3) | 0.134 | 476(63.4) | 479(63.8) | 0.838 | 1115(66.6) | 1135(67.8) | 0.704 |
| low to intermediate risk | 9423(30.9) | 9639(31.6) |  | 244(32.5) | 237(31.6) |  | 520(31.1) | 498(29.7) |  |
| high risk | 983(3.2) | 944(3.1) |  | 31(4.1) | 35(4.7) |  | 39(2.3) | 41(2.4) |  |
| Abbreviations: DTC=differentiated thyroid carcinoma; PSM=propensity score–matched; PTC=papillary thyroid carcinoma; OCA=oncocytic carcinoma of thyroid; FTC=Follicular thyroid carcinoma; RAI=Radioactive iodine; ATA=American Thyroid Association | | | | | | | | | |
