# Supplementary material for: Radioactive iodine therapy strategies for distinct types of differentiated thyroid cancer: a propensity score–matched analysis
Source: Front Endocrinol (Lausanne). 2023 Aug 17;14:1158581. doi: 10.3389/fendo.2023.1158581 (PMC10471126; doi:10.3389/fendo.2023.1158581)
Supplement: Supplementary file 3 [file Table_3.docx]

| **Supplementary Table. 3** Univariate analysis in PTC^[[1]](#footnote-1)^, OCA^[[2]](#footnote-2)^, and FTC^[[3]](#footnote-3)^ patients | | | | | | | | | | |
| --- | --- | --- | --- | --- | --- | --- | --- | --- | --- | --- |
| Variables | | Before PSM^[[4]](#footnote-4)^ | | | |  | After PSM | | | |
|  |  | HRs^[[5]](#footnote-5)^ | 95.0% CI^[[6]](#footnote-6)^ | | P |  | HRs | 95.0% CI | | P |
| **PTC** |  |  |  |  |  |  |  |  |  |  |
| Age | <55 | 1 | Reference | |  |  |  | Reference | |  |
|  | ≥55 | 8.012 | 6.994-9.178 | | <0.001 |  | 9.224 | 7.682-11.075 | | <0.001 |
| Sex | Female |  | Reference | |  |  |  | Reference | |  |
|  | Male | 2.897 | 2.599-3.229 | | <0.001 |  | 2.946 | 2.552-3.401 | | <0.001 |
| Race | White |  | Reference | | 0.088 |  |  | Reference | | 0.732 |
|  | Black | 0.964 | 0.756-1.23 | | 0.771 |  | 1.09 | 0.797-1.49 | | 0.589 |
|  | Other | 1.222 | 1.044-1.43 | | 0.013 |  | 1.118 | 0.904-1.384 | | 0.304 |
| T stage | T1 |  | Reference | |  |  |  | Reference | | <0.001 |
|  | T2 | 1.916 | 1.56-2.354 | | <0.001 |  | 2.075 | 1.603-2.686 | | <0.001 |
|  | T3 | 5.543 | 4.774-6.435 | | <0.001 |  | 6.418 | 5.27-7.816 | | <0.001 |
|  | T4 | 28.853 | 24.741-33.647 | | <0.001 |  | 35.609 | 29.035-43.671 | | <0.001 |
| N stage | N0 and Nx |  | Reference | |  |  |  | Reference | |  |
|  | N1 | 3.542 | 3.177-3.948 | | <0.001 |  | 4.107 | 3.56-4.739 | | <0.001 |
| M stage | M0 and Mx |  | Reference | |  |  |  | Reference | |  |
|  | M1 | 30.684 | 26.526-35.492 | | <0.001 |  | 37.739 | 31.014-45.923 | | <0.001 |
| ATA^[[7]](#footnote-7)^ Risk staging (TNM) | low risk |  | Reference | |  |  |  | Reference | | <0.001 |
|  | low to intermediate risk | 3.777 | 3.251-4.387 | | <0.001 |  | 4.27 | 3.521-5.179 | | <0.001 |
|  | high risk | 34.457 | 29.662-40.028 | | <0.001 |  | 42.352 | 34.896-51.402 | | <0.001 |
| Rad^[[8]](#footnote-8)^ | Yes |  | Reference | |  |  |  | Reference | |  |
|  | No | 0.645 | 0.576-0.723 | | <0.001 |  | 1.24 | 1.075-1.431 | | 0.003 |
| **FTC** |  |  |  |  |  |  |  |  |  |  |
| Age | <55 |  | Reference | |  |  |  | Reference | |  |
|  | ≥55 | 5.941 | 3.95-8.934 | | <0.001 |  | 5.75 | 3.367-9.819 | | <0.001 |
| Sex | Female |  | Reference | |  |  |  | Reference | |  |
|  | Male | 1.595 | 1.14-2.233 | | 0.006 |  | 1.651 | 1.046-2.607 | | 0.031 |
| Race | White |  | Reference | |  |  |  | Reference | |  |
|  | Black | 1.249 | 0.765-2.038 | | 0.374 |  | 0.987 | 0.489-1.993 | | 0.972 |
|  | Other | 1.939 | 1.233-3.049 | | 0.004 |  | 2.258 | 1.28-3.983 | | 0.005 |
| T stage | T1 |  | Reference | |  |  |  | Reference | |  |
|  | T2 | 2.396 | 1.104-5.199 | | 0.027 |  | 2.02 | 0.895-4.561 | | 0.091 |
|  | T3 | 7.519 | 3.628-15.584 | | <0.001 |  | 5.384 | 2.515-11.524 | | <0.001 |
|  | T4 | 49.783 | 22.679-109.278 | | <0.001 |  | 49.042 | 20.557-116.995 | | <0.001 |
| N stage | N0 and Nx |  | Reference | |  |  |  | Reference | |  |
|  | N1 | 11.39 | 7.745-16.75 | | <0.001 |  | 11.366 | 6.388-20.224 | | <0.001 |
| M stage | M0 and Mx |  | Reference | |  |  |  | Reference | |  |
|  | M1 | 25.307 | 17.806-35.967 | | <0.001 |  | 25.496 | 14.522-44.761 | | <0.001 |
| ATA Risk staging (TNM) | low risk |  | Reference | |  |  |  | Reference | |  |
|  | low to intermediate risk | 3.115 | 2.006-4.836 | | <0.001 |  | 3.031 | 1.806-5.087 | | <0.001 |
|  | high risk | 34.724 | 22.683-53.156 | | <0.001 |  | 34.262 | 19.64-59.769 | | <0.001 |
| Rad | Yes |  | Reference | |  |  |  | Reference | |  |
|  | No | 0.939 | 0.664-1.327 | | 0.72 |  | 1.253 | 0.811-1.934 | | 0.309 |
| **OCA** |  |  |  |  |  |  |  |  |  |  |
| Age | <55 |  | Reference | |  |  |  | Reference | |  |
|  | ≥55 | 4.51 | 2.708-7.51 | | <0.001 |  | 6.925 | 3.166-15.146 | | <0.001 |
| Sex | Female |  | Reference | |  |  |  | Reference | |  |
|  | Male | 1.959 | 1.334-2.877 | | 0.001 |  | 1.985 | 1.233-3.197 | | 0.005 |
| Race | White |  | Reference | |  |  |  | Reference | |  |
|  | Black | 0.717 | 0.291-1.764 | | 0.469 |  | 1.007 | 0.366-2.774 | | 0.989 |
|  | Other | 1.364 | 0.71-2.622 | | 0.352 |  | 1.316 | 0.568-3.049 | | 0.522 |
| T stage | T1 |  | Reference | |  |  |  | Reference | |  |
|  | T2 | 1.538 | 0.627-3.775 | | 0.347 |  | 1.263 | 0.459-3.475 | | 0.651 |
|  | T3 | 6.774 | 3.08-14.896 | | <0.001 |  | 6.678 | 2.827-15.774 | | <0.001 |
|  | T4 | 31.126 | 13.4-72.297 | | <0.001 |  | 23.924 | 9.191-62.277 | | <0.001 |
| N stage | N0 and Nx |  | Reference | |  |  |  | Reference | |  |
|  | N1 | 7.817 | 5.089-12.007 | | <0.001 |  | 7.016 | 4.093-12.025 | | <0.001 |
| M stage | M0 and Mx |  | Reference | |  |  |  | Reference | |  |
|  | M1 | 28.543 | 17.877-45.572 | | <0.001 |  | 26.567 | 12.476-56.576 | | <0.001 |
| ATA Risk staging (TNM) | low risk |  | Reference | |  |  |  | Reference | |  |
|  | low to intermediate risk | 6.433 | 3.544-11.675 | | <0.001 |  | 6.544 | 3.412-12.551 | | <0.001 |
|  | high risk | 47.589 | 25.785-87.83 | | <0.001 |  | 33.825 | 16.515-69.278 | | <0.001 |
| Rad | Yes |  | Reference | |  |  |  | Reference | |  |
|  | No | 0.982 | 0.655-1.474 | | 0.931 |  | 1.063 | 0.663-1.705 | | 0.799 |

1. PTC=papillary thyroid carcinoma [↑](#footnote-ref-1)
2. OCA=oncocytic carcinoma of thyroid [↑](#footnote-ref-2)
3. FTC=follicular thyroid carcinoma [↑](#footnote-ref-3)
4. PSM=propensity score–matched [↑](#footnote-ref-4)
5. HRs= Hazard ratios [↑](#footnote-ref-5)
6. CI= confidence index [↑](#footnote-ref-6)
7. ATA=American Thyroid Association [↑](#footnote-ref-7)
8. RAI=Radioactive iodine [↑](#footnote-ref-8)
